# Amyloid-β predominant Alzheimer’s disease neuropathologic change

**DOI:** 10.1093/brain/awae325

**Published:** 2024-10-17

**Authors:** Gabor G Kovacs, Yuriko Katsumata, Xian Wu, Khine Zin Aung, David W Fardo, Shelley L Forrest, Alzheimer's Disease Genetics Consortium, James D Bowen, Paul K Crane, Gail P Jarvik, C Dirk Keene, Eric B Larson, Wayne C McCormick, Susan M McCurry, Shubhabrata Mukherjee, Neil W Kowall, Ann C McKee, Robert A Stern, Clinton T Baldwin, Lindsay A Farrer, Gyungah Jun, Kathryn L Lunetta, Lawrence S Honig, Jean Paul Vonsattel, Jennifer Williamson, Scott Small, Sandra Barral, Christiane Reitz, Badri N Vardarajan, Richard Mayeux, James R Burke, Christine M Hulette, Kathleen A Welsh-Bohmer, Marla Gearing, James J Lah, Allan I Levey, Thomas S Wingo, Liana G Apostolova, Martin R Farlow, Bernardino Ghetti, Andrew J Saykin, Salvatore Spina, Kelley M Faber, Tatiana M Foroud, Marilyn S Albert, Constantine G Lyketsos, Juan C Troncoso, Matthew P Frosch, Robert C Green, John H Growdon, Bradley T Hyman, Rudolph E Tanzi, Huntington Potter, Dennis W Dickson, Nilufer Ertekin-Taner, Neill R Graff-Radford, Joseph E Parisi, Ronald C Petersen, Bradley F Boeve, Mariet Allen, Minerva M Carrasquillo, Steven G Younkin, Ranjan Duara, Joseph D Buxbaum, Alison M Goate, Mary Sano, Arjun V Masurkar, Thomas Wisniewski, Eileen H Bigio, Marsel Mesulam, Sandra Weintraub, Robert Vassar, Jeffrey A Kaye, Joseph F Quinn, Randall L Woltjer, Lisa L Barnes, Lei Yu, Denis A Evans, Victor Henderson, Kenneth B Fallon, Lindy E Harrell, Daniel C Marson, Erik D Roberson, Charles DeCarli, Lee-Way Jin, John M Olichney, Ronald Kim, Frank M LaFerla, Edwin Monuki, Elizabeth Head, David Sultzer, Daniel H Geschwind, Harry V Vinters, Marie-Francoise Chesselet, Douglas R Galasko, James B Brewer, Adam Boxer, Anna Karydas, Joel H Kramer, Bruce L Miller, Howard J Rosen, William W Seeley, Jeffrey M Burns, Russell H Swerdlow, Linda J Van Eldik, Roger L Albin, Andrew P Lieberman, Henry L Paulson, Steven E Arnold, John Q Trojanowski, Vivianna M Van Deerlin, Laura B Cantwell, Amanda P Kuzma, John Malamon, Adam C Naj, Liming Qu, Gerard D Schellenberg, Otto Valladares, Li-San Wang, Yi Zhao, Ronald L Hamilton, M Ilyas Kamboh, Oscar L Lopez, James T Becker, Chuanhai Cao, Ashok Raj, Amanda G Smith, Helena C Chui, Carol A Miller, John M Ringman, Lon S Schneider, Thomas D Bird, Joshua A Sonnen, Chang-En Yu, Thomas Grabowsk, Elaine Peskind, Murray Raskind, Ge Li, Debby W Tsuang, Sanjay Asthana, Craig S Atwood, Cynthia M Carlsson, Mark A Sager, Nathaniel A Chin, Suzanne Craft, Nigel J Cairns, John C Morris, Carlos Cruchaga, Stephen Strittmatter, Eric M Reiman, Thomas G Beach, Matthew J Huentelman, John Hardy, John S K Kauwe, Hakon Hakonarson, Deborah Blacker, Thomas J Montine, William S Bush, Jonathan L Haines, Alan J Lerner, Xiongwei Zhou, Gary W Beecham, Regina M Carney, Michael L Cuccaro, John R Gilbert, Kara L Hamilton-Nelson, Brian W Kunkle, Eden R Martin, Margaret A Pericak-Vance, Jeffery M Vance, Amanda J Myers, James B Leverenz, Philip L De Jager, Mindy J Katz, Richard B Lipton, Valory Pavlik, Paul Massman, Eveleen Darby, Monica Rodriguear, Aisha Khaleeq, Donald R Royall, Alan Stevens, Marcia Ory, John C DeToledo, Henrick Wilms, Kim Johnson, Victoria Perez, Michelle Hernandez, Kirk C Wilhelmsen, Jeffrey Tilson, Scott Chasse, Robert C Barber, Thomas J Fairchild, Sid E O’Bryant, Janice Knebl, James R Hall, Leigh Johnson, Douglas Mains, Lisa Alvarez, Adriana Gamboa, David Paydarfar, John Bertelson, Martin Woon, Gayle Ayres, Alyssa Aguirre, Raymond Palmer, Marsha Polk, Perrie M Adams, Ryan M Huebinger, Joan S Reisch, Roger N Rosenberg, Munro Cullum, Benjamin Williams, Mary Quiceno, Linda Hynan, Janet Smith, Barb Davis, Trung Nguyen, Ekaterina Rogaeva, Peter St George-Hyslop, Peter T Nelson

**Affiliations:** Tanz Centre for Research in Neurodegenerative Disease and Department of Laboratory Medicine and Pathobiology, University of Toronto, Toronto, Ontario M5T 0S8, Canada; Laboratory Medicine Program and Krembil Brain Institute, University Health Network, Toronto, Ontario M5G 2C4, Canada; Department of Biostatistics, University of Kentucky, Lexington, KY 40536-0679, USA; Sanders-Brown Center on Aging, University of Kentucky, Lexington, KY 40536, USA; Department of Biostatistics, University of Kentucky, Lexington, KY 40536-0679, USA; Sanders-Brown Center on Aging, University of Kentucky, Lexington, KY 40536, USA; Department of Biostatistics, University of Kentucky, Lexington, KY 40536-0679, USA; Sanders-Brown Center on Aging, University of Kentucky, Lexington, KY 40536, USA; Department of Biostatistics, University of Kentucky, Lexington, KY 40536-0679, USA; Sanders-Brown Center on Aging, University of Kentucky, Lexington, KY 40536, USA; Tanz Centre for Research in Neurodegenerative Disease and Department of Laboratory Medicine and Pathobiology, University of Toronto, Toronto, Ontario M5T 0S8, Canada; Laboratory Medicine Program and Krembil Brain Institute, University Health Network, Toronto, Ontario M5G 2C4, Canada; Sanders-Brown Center on Aging, University of Kentucky, Lexington, KY 40536, USA; Department of Pathology, Division of Neuropathology, University of Kentucky, Lexington, KY 40536-0679, USA

**Keywords:** amyloid-β, biomarker, NACC, ADGC, diffuse plaques

## Abstract

Different subsets of Alzheimer’s disease neuropathologic change (ADNC), including the intriguing set of individuals with severe/widespread amyloid-β (Aβ) plaques but no/mild tau tangles [Aβ-predominant (AP)-ADNC], may have distinct genetic and clinical features.

Analysing National Alzheimer’s Coordinating Center data, we stratified 1187 participants into AP-ADNC (*n* = 95), low Braak primary age-related tauopathy (PART; *n* = 185), typical-ADNC (*n* = 832) and high-Braak PART (*n* = 75). AP-ADNC differed in some clinical features and genetic polymorphisms in the *APOE*, *SNX1*, *WNT3*/*MAPT* and *IGH* genes.

We conclude that AP-ADNC differs from classical ADNC with implications for *in vivo* studies.

## Teaser

Kovacs *et al.* report that autopsy-confirmed cases of amyloid-β predominant Alzheimer’s disease neuropathologic change (AP-ADNC)—marked by severe/widespread amyloid-β plaques but no/mild tau tangles—differ in clinical and genetic features from classical ADNC (amyloid-β and tau), with implications for *in vivo* studies.

## Introduction

The availability of Alzheimer’s disease (AD) therapeutics has focused intense attention on the potential for disease-specific biomarkers and outcome prediction. AD neuropathologic change (ADNC) is classically characterized by the concomitant presence of amyloid-β (Aβ) and tau pathologies.^1^ Neuropathological studies suggest that Aβ deposition begins in the neocortex, followed by the hippocampus and affects the striatum, brainstem, and cerebellum in hierarchical order as the disease becomes more advanced.^2^ In contrast, tau pathology (neurofibrillary tangles, NFTs) affects the brainstem early, followed by the limbic and neocortical areas.^3^

According to the prevailing Amyloid Cascade Hypothesis,^4^ Aβ deposition tends to potentiate tau pathology. However, some brains with widespread/severe Aβ deposition (in cortex, brainstem and cerebellum) have none, or quite mild, tau pathology (Braak NFT stages 0–II).^5,6^ These intriguing ‘mis-match’ Aβ+/tau− cases, which we term amyloid-predominant ADNC (AP-ADNC), have implications about clinical management and disease (and disease-resistance) mechanisms and are the focal point of this article. Analysing data from the large and granular National Alzheimer’s Coordinating Center (NACC) dataset^7^ and the Alzheimer’s Disease Genetics Consortium (ADGC),^8^ we present here genetic and clinical observations on individuals with autopsy-confirmed AP-ADNC status, providing insights into the causes and consequences of various Aβ and tau pathologic combinations in aged human brains.

## Patients and methods

### Participants

Clinical and neuropathological data were derived from the NACC Uniform Dataset (UDS) and Neuropathology (NP) September 2022 data freeze; these came from the National Institute of Health/National Institute on Aging-funded Alzheimer’s Disease Research Centers (ADRCs).^7^ Each ADRC obtained written informed consent from their participants with Institutional Review Board review and approval.

### Data from NACC: pathologic and clinical

Our goals were to understand common pathologic, clinical and genetic phenomena; therefore participants were excluded if diagnosed with any of 27 rare brain diseases (Supplementary Table 1). Also excluded were participants who had missing data for Thal Aβ phase and/or Braak NFT stage.

Four groups were defined based on Thal Aβ phase and Braak NFT stage status^1^ (Supplementary Table 2): (i) AP-ADNC = Thal Aβ phase 4–5 and Braak NFT stage 0–II; (ii) typical-ADNC = Thal Aβ phase 4–5 and Braak NFT stage III–V (ADNC intermediate or high); (iii) PART-low = Thal Aβ phase 0 and Braak NFT stage 0–II; and (iv) PART-high = Thal Aβ phase 0 and Braak NFT stage III–IV.

The rationale for the grouping was to compare AP-ADNC cases with those showing only tau pathology in early and fully developed forms (i.e. PART-low and -high) and with intermediate or high typical-ADNC. The latter group represents the cases mostly seen in clinical practice; therefore, we excluded cases with Braak NFT stage VI. As an additional evaluation, we performed further comparisons in which Braak NFT stage VI cases were included in the typical-ADNC group.

Limbic-predominant age-related TDP-43 encephalopathy neuropathologic change (LATE-NC), Lewy body pathology, arteriolosclerosis, and infarcts and lacunes were operationalized according to pathologic features, as described previously.^9^

Cognitive data included Mini-Mental State Examination (MMSE) and Montreal Cognitive Assessment (MoCA) for global function; Animal Naming and Boston Naming for language/fluency function; Wechsler Memory Scale-Revised (WMS-R) Logical Memory—immediate and delayed and Craft Story 21 Recall—immediate and delayed for memory function; and Digit Span forward and backward for working memory.^9^ Since the MoCA and Craft Story 21 Recall—immediate and delayed were introduced in the NACC UDS version 3 from March 2015 instead of MMSE and WMS-R Logical Memory—immediate and delayed, respectively, we transformed the new battery scores into equivalent old battery scores based on Monsell and colleagues’ crosswalk study.^10^ We also evaluated the Clinical Dementia Rating Scale (CDR) Sum of Boxes ratings. Neuropsychiatric symptoms were operationalized using the Neuropsychiatric Inventory (NPI-Q).^11^ Study co-participants were asked if symptoms were present in the month prior to the study visit. All clinical data were measured at last visit within 3 years before death.

### Genetic data

Genetic data were obtained from ADGC, which were linked to the NACC UDS and NP dataset as described previously.^8^ The genotype data were imputed using the TOPMed Imputation Server (https://imputation.biodatacatalyst.nhlbi.nih.gov/) based on GRCh38. We examined 84 AD-related single nucleotide polymorphisms (SNPs) that were reported to be associated with AD-type (mostly clinically operationalized) dementia, by Bellenguez *et al*.,^12^ and two SNPs in *APOE* (rs429358 and rs7412). When SNPs were missing, we used the proxies which were in perfect linkage disequilibrium (LD; r2 = 1 and D′ = 1) identified using LDLink^13^ (ldlink nih.gov) by querying the LDproxy Tool, in which we selected EUR in GRCh38 using a ±5000 bp window to determine the proxies based on their highest correlations.

### Statistical analysis

A logistic regression model was run for each of the above-mentioned neuropathologies and each of the neuropsychiatric symptoms, and analysis of covariance for each of the cognitive test scores to examine associations with the AP-ADNC group. These models had clinical and other neuropathological variables as an outcome and the AP-ADNC group as a predictor and were adjusted for sex, age at death and *APOE* ε4 allele count. The genetic associations with the AP-ADNC groups were examined using a multinomial regression model adjusted for sex and age at death, and the top three principal components were computed in PLINK v1.90a under an additive mode of inheritance. We confirmed that participants were genetically independent using estimated proportion identity-by-descent (all < 0.185).

## Results

After exclusions were applied, 1187 participants were categorized into four groups: *n* = 95 in group AP-ADNC; *n* = 832 in group typical-ADNC (intermediate and high but excluding Braak stage VI); *n* = 185 in group PART-low; and *n* = 75 in group PART-high (Supplementary Fig. 1). Selected characteristics of each group are displayed in Supplementary Table 3. *APOE* ε4 was associated with classic ADNC, moreso than with AP-ADNC [odds ratio (OR) = 1.80 and *P*-value = 0.004].

Compared with group AP-ADNC, group PART-low had lower risk of Lewy body pathology and group PART-high had lower risk of brain arteriolosclerosis (Table 1 and Supplementary Table 4). The percentage of cognitively unimpaired was 36.8% (*n* = 35) for AP-ADNC, 9.7% (*n* = 81) for typical-ADNC, 38.3% (*n* = 71) for PART-low and 38.7% (*n* = 29) for PART-high.

**Table 1:** Associations between neuropathologies and AP-ADNC versus other groups

Group^a^	Adjusted^b^		
	OR	95% CI	*P*-value
LATE-NC			
Typical-ADNC	1.52	0.79–2.90	0.21
PART-low	0.78	0.34–1.80	0.56
PART-high	0.38	0.11–1.26	0.11
Lewy bodies			
Typical-ADNC	1.16	0.63–2.14	0.11
PART-low	**0**.**44**	**0.20–0.98**	**0**.**043**
PART-high	0.66	0.23–1.89	0.21
Arteriolosclerosis			
Typical-ADNC	0.81	0.43–1.54	0.53
PART-low	0.49	0.22–1.12	0.092
PART-high	**0**.**16**	**0.03–0.74**	**0**.**019**
Infarcts and lacunes			
Typical-ADNC	0.93	0.51–1.69	0.81
PART-low	1.23	0.61–2.45	0.56
PART-high	0.85	0.36–2.03	0.72

AP-ADNC = Thal phase 4–5 and Braak NFT stage 0–2, Typical-ADNC = Thal phase 4–5 and Braak NFT stage 3–5, PART-low = Thal phase 0 and Braak NFT stage 0–2, PART-high = Thal phase 0 and Braak NFT stage 3–4. LATE-NC was defined as TDP-43 immunoreactive inclusion (NPTDPC) ‘Yes’ in hippocampus; Lewy body disease (NACCLEWY) was dichotomized as 0 = No Lewy body pathology and 1 = Lewy body pathology in any brain region; arteriolosclerosis (NACCARTE) was dichotomized as 0 = none/mild/moderate and 1 = severe; the original scale for infarcts and lacunes is 0 = No and 1 = Yes. *P <* 0.05 is highlighted in bold. ADNC = Alzheimer’s disease neuropathologic change; AP = amyloid- predominant; CI = confidence interval; LATE-NC = limbic-predominant age-related TDP-43 encephalopathy neuropathologic change; OR = odds ratio; PART = primary age-related tauopathy.

^a^AP-ADNC was the reference group.

^b^Adjusted for the number of *APOE* ε4, age at death, and sex.

The CDR Sum of Boxes ratings, MMSE, Boston Naming and Logical Memory immediate and delayed scores were lower in the typical-ADNC group than all other groups (Supplementary Fig. 2). Compared with the AP-ADNC group, the typical-ADNC group had higher risk of apathy or indifference and lower risk of nighttime behaviours, and the PART-low group had lower risk of appetite and eating problems (Supplementary Tables 5 and 6).

SNPs rs3848143 in *SNX1* and rs199515 in *WNT3* were also associated with the AP-ADNC group. Notably, the AP-ADNC-related *WNT3* variant is in LD with other genetic variants that are proxies for the *MAPT* haplotype (rs9468 and rs199515; r2 = 0.79 and D′ = 0.91). This *WNT3* SNP was nominally different between the AP-ADNC and typical-ADNC groups (OR = 0.64 and *P*-value = 0.031) (Table 2 and Supplementary Tables 7 and 8).

**Table 2:** Genetic associations with group in the multinomial regression analyses (AP-ADNC versus other groups)

Variant	Gene	Typical-ADNC			PART-low			PART-high		
		OR	SE	*P*	OR	*SE*	*P*	OR	SE	*P*
rs679515	*CR1*	0.77	0.22	0.24	0.58	0.28	**0**.**045**	0.61	0.33	0.14
rs6733839	*BIN1*	1.40	0.19	0.077	1.14	0.22	0.55	1.70	0.26	**0**.**041**
rs113706587	*RASGEF1C*	0.68	0.27	0.15	0.53	0.33	0.057	0.38	0.45	**0**.**032**
rs10947943	*UNC5CL*	1.36	0.29	0.29	1.46	0.33	0.25	2.07	0.36	**0**.**044**
rs7767350	*CD2AP*	0.80	0.19	0.22	0.60	0.23	**0**.**030**	0.83	0.27	0.48
rs7157106	*IGH gene cluster*	1.68	0.21	**0**.**015**	1.31	0.25	0.27	1.99	0.28	**0**.**013**
rs10131280	*IGH gene cluster*	1.75	0.33	0.089	2.76	0.36	**0**.**0048**	2.23	0.41	**0**.**048**
rs3848143	*SNX1*	2.32	0.28	**0**.**0025**	2.28	0.31	**0**.**0076**	2.80	0.34	**0**.**0026**
rs56407236	*PRDM7*	0.60	0.29	0.081	0.71	0.35	0.33	0.34	0.53	**0**.**041**
rs2242595	*MYO15A*	0.73	0.25	0.21	0.85	0.30	0.57	0.33	0.46	**0**.**016**
rs199515	*WNT3*	0.64	0.21	**0**.**031**	0.74	0.25	0.24	0.75	0.29	0.33
rs429358	*APOE*	1.94	0.24	**0**.**0059**	0.21	0.37	**2.5 × 10^−5^**	–	–	–
rs7412	*APOE*	0.33	0.32	**5.5 × 10^−4^**	1.06	0.35	0.86	1.27	0.40	0.54

AP-ADNC is the reference group. *P <* 0.05 is highlighted in bold. AP-ADNC = Thal phase 4–5 and Braak NFT stage 0–2; Typical-ADNC = Thal phase 4–5 and Braak NFT stage 3–5; PART-low = Thal phase 0 and Braak NFT stage 0–2; PART-high = Thal phase 0 and Braak NFT stage 3–4. ADNC = Alzheimer’s disease neuropathologic change; AP = amyloid- predominant; CI = confidence interval; OR = odds ratio; PART = primary age-related tauopathy; SE = standard error.

## Discussion

Clinicians managing patients at risk for cognitive impairment in ageing will likely experience AP-ADNC patients. Biomarkers that reflect the underlying pathology, even at the preclinical stage, are central considerations for ADNC-oriented clinical management and clinical trials. A commonly used approach is the biomarker-based assessment of Aβ *(*A), pathological tau (T) and neurodegeneration (N) markers.^14^ This recognizes A(+)T(+)N(+/−) as predicting AD and A(−)T(+)N(+/−) as non-AD pathologic change. Patients with an A(+)T(−)N(−) biomarker profile are interpreted in the framework of the AD continuum, suggesting an early phase of Aβ pathology where pathological tau has not yet reached the threshold for biomarker detection.^14^ This concept has been expanded to include core 1 and core 2 biomarkers.^15^ Core 1 biomarkers reflect ADNC more generally (i.e. neuritic plaques and neurofibrillary tangles) and assume that biomarkers of Aβ and phosphorylated and secreted soluble tau become abnormal about the same time, while biomarkers of insoluble tau aggregates (core 2, which includes tau PET and biofluid tau MTBR243) become abnormal later, closer in time to the onset of overt clinical symptoms.^15^ Here, using the relatively large and data-rich dataset of the NACC and ADGC, we report some genetic features and clinical symptoms that may help to distinguish AP-ADNC from typical-ADNC or PART. Regarding biomarkers, AP-ADNC might lack or have low levels of markers of insoluble tau aggregates, but autopsy-confirmed studies are needed to evaluate the behaviour of phosphorylated and secreted soluble tau as compared to cases with typical-ADNC.

Pathological patterns similar to what we are proposing as AP-ADNC have been described before. ‘Plaque-only dementia’ is a term that refers to cases with amyloid plaques without tangles in the cortex; its use predates the current ADNC concept and belies the fact that many persons with Aβ plaques but lacking substantial tangles are not demented.^5^ Another term used in the literature to describe cases with significant Aβ deposits in neocortical and/or limbic areas in the setting of minimal neurofibrillary pathology is ‘pathological ageing’.^16^ An implication of the term pathological ageing is that the affected person was cognitively normal before death.^17^ The term AP-ADNC disambiguates the concept and acknowledges that with or without cognitive impairment, these cases belong on the ADNC pathological spectrum—a brain with Aβ plaques is, by definition, at least low ADNC.^1^ This concept is also significant for ‘A’-type amyloidosis when diagnosing live individuals using biomarkers. AP-ADNC indicates cases with Aβ deposits involving cortical, subcortical, brainstem and cerebellum and can be seen at autopsy in persons who either did or did not show cognitive decline during life (∼63% showed cognitive decline in the present study). The severity of cognitive impairment may be related to a number of causes, including the presence of co-pathologies, as well as to phenotypic characteristics in the profile of brain fibrillar and soluble Aβ, glial response, synaptic dysfunction or differences in cerebral blood flow.^18,19^

Recent studies suggest that phosphorylated-tau^20^ and plasma brain-derived tau^21^ measurements can reflect the Aβ pathology in the brain. The constellation of less neuritic plaques and NFTs and widespread Aβ deposition as in AP-ADNC has to be translated into the biomarker practice. It will be interesting to re-evaluate cohorts with these measurements and compare with Aβ-based biomarkers to identify this subgroup *in vivo*. A recent PET-based study showed that the magnitude and topography of tau deposition were closely related to the duration of amyloid deposition in preclinical and symptomatic individuals and supported the notion that the combination of Aβ and tau is required for accelerated longitudinal cognitive decline in preclinical AD.^22^ The same study showed, however, that individuals with mild cognitive impairment can have Aβ positivity with low or no PET stage of tau deposition; furthermore, this discrepancy was seen in a few cases with dementia.^22^

We confirmed^23^ that *APOE* ε4 is not the driving force to develop AP-ADNC as it is for typical-ADNC. Our study also provides new insights into the associations between ADNC-related phenomena and SNPs in the *SNX1* and *IGH* genes.^12,24^  *SNX1* is among a group of proteins performing cargo sorting at the endosome,^25^ and human *IGH* seems to have inherent anti-amyloidogenic activity,^26^ so may have a differential role in Aβ and tau pathogenesis. These SNPs, together with *WNT3* gene variants, helped distinguish AP-ADNC from typical-ADNC. In addition to WNT being one of the signalling pathways involved in brain development and those related to AD,^27^ SNPs in *WNT3* are proxies of the *MAPT* haplotypes H1 and H2, suggesting resilience against developing tau pathology.^28^ Recent studies have highlighted the involvement of WNT3a in the protective pattern from early tau phosphorylation associated with the resistance to familial AD.^29^ In the future, genetic assessments may augment other predictive biomarkers to help indicate patients’ risk and to sharpen personalized medical care.

Limitations of the study include challenges in the population representativeness of autopsy cohorts, and specifically as applies to the NACC neuropathology dataset: all 20 ADRCs from which cases were obtained have some overlapping biases, including exclusion criteria (e.g. lack of substance abuses, lack of some neuropsychiatric changes) or relative paucity of Blacks and Latinx populations that make the research participants studied not representative of a broader population.^30,31^ Furthermore, the academic clinics from which these cases derived were ‘AD research centres’; thus, both the clinics and the participants themselves may be selected based on interest in AD/amnestic dementia and may overlook some other common dementia types.^30^

**Figure 1:**
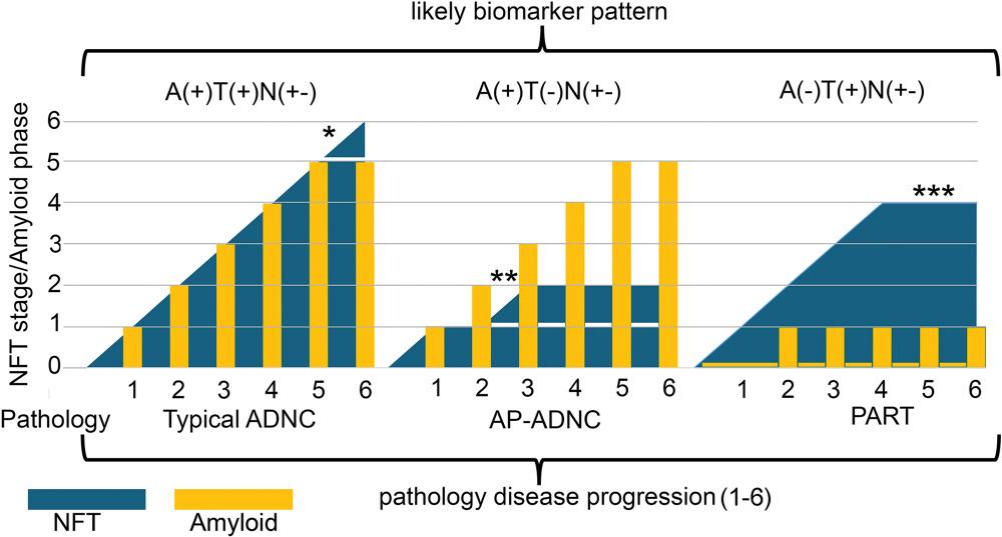
**Conceptual summary of the spectrum of Alzheimer’s disease-related pathologies.** Alzheimer’s disease (AD)-related pathologies include the presence of neurofibrillary tangles (NFTs) in six stages and the presence of amyloid-β (Aβ) plaques in five phases.^1^ Plotting these variables on a six-tiered disease progression scale also representing the NFT stages revealed major differences between typical AD neuropathologic change (NC), Aβ predominant ADNC (AP-ADNC) and primary age-related tauopathy (PART). These are associated with different *in vivo* biomarker patterns reflecting the Aβ amyloid (A) pathological Tau (T) and neurodegeneration (N) states.^14^ *In typical-ADNC, the highest NFT stage and Aβ phase is 6 and 5, respectively. **In AP-ADNC, the NFT stage does not increase beyond 2. ***In PART Aβ, phase does not increase beyond 1.

In summary, the present study helps fill out the spectrum of common combinations of Aβ and tau proteinopathies in aged brains. Analogous to PART (tau only), AP-ADNC differs from the classical AD continuum (Aβ and tau) (Fig. 1), based on correlated genetics and clinical findings. We propose that AP-ADNC is not simply ‘pathological ageing’ and, similarly to typical-ADNC, further studies are needed to evaluate what factors are associated with cognitive decline in individuals with AP-ADNC. Biomarker studies should identify AP-ADNC *in vivo* to facilitate risk stratification and identify pathways specific for typical-ADNC, AP-ADNC and PART.

## Supplementary Material

awae325_Supplementary_Data

## Data Availability

The data that support the findings of this study are available from the corresponding author upon reasonable request.
