# Supplementary material for: Amyloid-β predominant Alzheimer’s disease neuropathologic change
Source: Brain. 2024 Oct 17;148(2):401–7. doi: 10.1093/brain/awae325 (PMC11788189; doi:10.1093/brain/awae325)
Supplement: awae325_Supplementary_Data [file awae325_supplementary_data.pdf]

Gabor G. Kovacs, Yuriko Katsumata, Xian Wu, Khine Zin Aung, Shelley L. Forrest, Peter T. Nelson

Supplementary Table 1. Exclusion criteria in the National Alzheimer's Coordination Center Neuropathology Form.

Exclusion criteria	Variable name
Down syndrome	NACCDOWN
Pigment-spheroid degeneration/NBIA	NPPDXA
Multiple system atrophy	NPPDXB
Trinucleotide disease (Huntington disease, SCA, other)	NPPDXD
Malformation of cortical development	NPPDXE
Metabolic/storage disorder of any type	NPPDXF
White matter disease, leukodystrophy	NPPDXG
White matter disease, multiple sclerosis or other demyelinating disease	NPPDXH
Prion disease	NACCPRIO
FTLD-tau	NPFTDTAU
ALS/motor neuron disease (MND)	NPALSMND
CADASIL	NPPATH10
FTLD with TDP-43 pathology (FTLD-TDP)	NPFTDTDP
Other FTLD	NPOFTD
FTLD-tau subtype — Pick's (PiD)	NACCPICK
FTLD-tau subtype — other 3R tauopathy	NPFTDT2
FTLD-tau subtype — corticobasal degeneration (CBD)	NACCCBD
FTLD-tau subtype — progressive supranuclear palsy (PSP)	NACCPROG
FTLD-tau subtype — argyrophilic grains	NPFTDT5
FTLD-tau subtype — other 4R tauopathy	NPFTDT6
FTLD-tau subtype — chronic traumatic encephalopathy	NPFTDT7
FTLD-tau subtype — amyotrophic lateral sclerosis	NPFTDT8
FTLD-tau subtype — tangle dominant disease	NPFTDT9
FTLD-tau subtype — other 3R + 4R tauopathy	NPFTDT10
Frontemporal dementia and parkinsonism with tau-positive or argyrophilic inclusions	NPFRONT
Tauopathy, other	NPTAU
FTD with ubiquitin-positive (tau-negative) inclusions	NPFTD

Supplementary Table 2. Grouping criteria.

Group	Thal Phase	Braak NFT Stage	n
AP-ADNC	4 or 5	0 to 2	95
Typical-ADNC	4 or 5	3 to 5	832
PART-low	0	0 to 2	185
PART-high	0	3 to 4	75

NFT = neurofibrillary tangle; AP-ADNC = Amyloid-Predominant Alzheimer's disease neuropathologic change; PART = primary age-related tauopathy

Supplementary Table 3. Characteristics of study participants.

Characteristics	Overall	Group			
		AP-ADNC	Typical ADNC	PART-low	PART-high
	n = 1,187	n = 95	n = 832	n = 185	n = 75
Age at death, mean ± SD	85.0 ± 9.9	85.2 ± 9.9	85.4 ± 9.2	81.4 ± 12.2	88.9 ± 8.6
Years in education, mean ± SD	16.4 ± 7.5	16.3 ± 2.5	16.2 ± 7.2	16.6 ± 9.2	17.6 ± 10.1
Sex, n (%)					
Male	660 (55.6)	52 (54.7)	455 (54.7)	113 (61.1)	40 (53.3)
Female	527 (44.4)	43 (45.3)	377 (45.3)	72 (38.9)	35 (46.7)
*APOE**, n (%)					
_/_	631 (53.2)	54 (56.8)	366 (44)	145 (78.4)	66 (88)
_/ε4	355 (29.9)	28 (29.5)	311 (37.4)	15 (8.1)	1 (1.3)
ε4/ ε4	67 (5.6)	2 (2.1)	64 (7.7)	1 (0.5)	0 (0)
Missing	134 (11.3)	11 (11.6)	91 (10.9)	24 (13)	8 (10.7)
Neuritic plaques, n (%)					
No	317 (26.7)	31 (32.6)	26 (3.1)	185 (100)	75 (100)
Sparse	152 (12.8)	33 (34.7)	119 (14.3)	0 (0)	0 (0)
Moderate	285 (24)	21 (22.1)	264 (31.7)	0 (0)	0 (0)
Frequent	433 (36.5)	10 (10.5)	423 (50.8)	0 (0)	0 (0)
Missing	0 (0)	0 (0)	0 (0)	0 (0)	0 (0)
LATE-NC, n (%)					
No	661 (55.7)	58 (61.1)	439 (52.8)	119 (64.3)	45 (60.0)
Yes	218 (18.4	14 (14.7)	184 (22.1)	16 (8.6)	4 (5.3)
Missing	308 (25.9)	23 (24.3)	209 (25.1)	50 (27)	26 (34.7)
Lewy bodies, n (%)					
No	717 (60.4)	60 (63.2)	454 (54.6)	146 (78.9)	57 (76)
Others	248 (20.9)	19 (20)	198 (23.8)	21 (11.4)	10 (13.3)
Neocortex	216 (18.2)	16 (16.8)	175 (21)	17 (9.2)	8 (10.7)
Missing	6 (0.5)	0 (0)	5 (0.6)	1 (0.5)	0 (0)

SD = standard deviation; LATE-NC = limbic-predominant age-related TDP-43 encephalopathy neuropathologic change.
AP-ADNC = Thal phase 4-5 and Braak NFT stage 0-2, Typical ADNC = Thal phase 4-5 and Braak NFT stage 3-5, PART-low = Thal phase 0 and Braak NFT stage 0-2, PART-high = Thal phase 0 and Braak NFT stage 3-4
*Typical-ADNC group had 1.8 times larger odds for *APOE* ε4 than AP-ADNC group (OR = 1.80 and p-value = 0.0044).

Supplementary Table 3. (Continued).

Characteristics	Overall	Group			
		AP-ADNC	Typical ADNC	PART-low	PART-high
	n = 1,187	n = 95	n = 832	n = 185	n = 75
Arteriolosclerosis, n (%)					
None	178 (15)	13 (13.7)	110 (13.2)	43 (23.2)	12 (16)
Mild	429 (36.1)	34 (35.8)	290 (34.9)	74 (40)	31 (41.3)
Moderate	389 (32.8)	29 (30.5)	292 (35.1)	41 (22.2)	27 (36)
Severe	147 (12.4)	16 (16.8)	108 (13)	21 (11.4)	2 (2.7)
Missing	44 (3.7)	3 (3.2)	32 (3.8)	6 (3.2)	3 (4)
Cerebral amyloid angiopathy, n (%)					
None	491 (41.4)	41 (43.2)	211 (25.4)	173 (93.5)	66 (88)
Mild	341 (28.7)	26 (27.4)	301 (36.2)	7 (3.8)	7 (9.3)
Moderate	227 (19.1)	16 (16.8)	205 (24.6)	5 (2.7)	1 (1.3)
Severe	127 (10.7)	12 (12.6)	114 (13.7)	0 (0)	1 (1.3)
Missing	1 (0.1)	0 (0)	1 (0.1)	0 (0)	0 (0)
Infarcts and lacunes, n (%)					
No	973 (82)	75 (78.9)	691 (83.1)	147 (79.5)	60 (80)
Yes	207 (17.4)	18 (18.9)	138 (16.6)	38 (20.5)	13 (17.3)
Missing	7 (0.6)	2 (2.1)	3 (0.4)	0 (0)	2 (2.7)

AP-ADNC = Thal phase 4-5 and Braak NFT stage 0-2, Typical ADNC = Thal phase 4-5 and Braak NFT stage 3-5, PART-low = Thal phase 0 and Braak NFT stage 0-2, PART-high = Thal phase 0 and Braak NFT stage 3-4

Supplementary Table 4. Associations between other neuropathologies and group AP-ADNC versus other groups in sensitivity analyses in which group typical ADNC included Braak stage VI.

Group*	Adjusted**		
	OR	95 % CI	P-value
LATE-NC			
Typical-ADNC	1.77	0.94 – 3.33	0.079
PART-low	0.74	0.32 – 1.70	0.48
PART-high	0.38	0.11 – 1.27	0.12
Lewy bodies			
Typical-ADNC	0.72	0.40 – 1.30	0.28
PART-low	0.46	0.21 – 1.30	0.055
PART-high	0.58	0.21 – 1.63	0.30
Arteriolosclerosis			
Typical-ADNC	0.92	0.49 – 1.70	0.78
PART-low	0.59	0.26 – 1.33	0.20
PART-high	**0.18**	**0.04 – 0.81**	**0.026**
Infarcts and lacunes			
Typical-ADNC	0.81	0.45 – 1.46	0.49
PART-low	1.36	0.68 – 2.71	0.38
PART-high	0.88	0.37 – 2.10	0.78

OR = odds ratio; CI = confidence interval; LATE-NC = limbic-predominant age-related TDP-43 encephalopathy neuropathologic change
* AP-ADNC was the reference group.
** Adjusted for the number of *APOE* ε4, age at death, and sex.
P< 0.05 is highlighted with bold letters.

AP-ADNC = Thal phase 4-5 and Braak NFT stage 0-2, Typical ADNC = Thal phase 4-5 and Braak NFT stage 3-5, PART-low = Thal phase 0 and Braak NFT stage 0-2, PART-high = Thal phase 0 and Braak NFT stage 3-4

LATE-NC was defined as TDP-43 immunoreactive inclusion (NPTDPC) "Yes" in hippocampus; Lewy body disease (NACCLEWY) was dichotomized as 0 = No Lewy body pathology and 1 = Lewy body pathology in any brain region; arteriolosclerosis (NACCARTE) was dichotomized as 0 =None/Mild/Moderate and 1 = Severe; the original scale for Infarcts and lacunes is 0 = No and 1 = Yes.

Supplementary Table 5. Frequency of neuropsychiatric symptoms at the last visit within three years before death by groups.

Symptom	Group, n (%)			
	AP-ADNC	Typical ADNC	PART-low	PART-high
Delusions				
No	68 (85.0)	493 (78.5)	128 (88.3)	51 (91.1)
Yes	12 (15.0)	135 (21.5)	17 (11.7)	5 (8.9)
Hallucinations				
No	65 (81.2)	514 (82)	125 (86.2)	50 (89.3)
Yes	15 (18.8)	113 (18)	20 (13.8)	6 (10.7)
Agitation or aggression				
No	56 (70.0)	407 (63.7)	108 (75)	43 (76.8)
Yes	24 (30.0)	232 (36.3)	36 (25)	13 (23.2)
Depression or dysphoria				
No	52 (65.8)	407 (64.4)	87 (61.3)	34 (61.8)
Yes	27 (34.2)	225 (35.6)	55 (38.7)	21 (38.2)
Anxiety				
No	60 (75.0)	392 (61.6)	106 (73.1)	39 (69.6)
Yes	20 (25.0)	244 (38.4)	39 (26.9)	17 (30.4)
Elation or euphoria				
No	78 (97.5)	611 (95.6)	143 (98.6)	56 (100)
Yes	2 (2.5)	28 (4.4)	2 (1.4)	0 (0)
Apathy or indifference				
No	56 (70.0)	351 (55.4)	92 (63.4)	33 (58.9)
Yes	24 (30.0)	283 (44.6)	53 (36.6)	23 (41.1)
Disinhibition				
No	66 (83.5)	490 (76.9)	126 (86.9)	48 (85.7)
Yes	13 (16.5)	147 (23.1)	19 (13.1)	8 (14.3)
Irritability or lability				
No	58 (72.5)	398 (62.3)	97 (66.9)	41 (73.2)
Yes	22 (27.5)	241 (37.7)	48 (33.1)	15 (26.8)
Motor disturbance				
No	70 (88.6)	453 (71.2)	122 (85.3)	52 (94.5)
Yes	9 (11.4)	183 (28.8)	21 (14.7)	3 (5.5)
Nighttime behaviors				
No	43 (56.6)	415 (66.3)	91 (65.0)	39 (73.6)
Yes	33 (43.4)	211 (33.7)	49 (35.0)	14 (26.4)
Appetite and eating problems				
No	56 (71.8)	433 (67.9)	99 (68.8)	39 (69.6)
Yes	22 (28.2)	205 (32.1)	45 (31.2)	17 (30.4)

AP-ADNC = Thal phase 4-5 and Braak NFT stage 0-2, Typical ADNC = Thal phase 4-5 and Braak NFT stage 3-5, PART-low = Thal phase 0 and Braak NFT stage 0-2, PART-high = Thal phase 0 and Braak NFT stage 3-4

Supplementary Table 6. Association between groups and neuropsychiatric symptoms at the last visit within three years before death by groups.

Group*	Adjusted**		
	OR	95 % CI	P-value
Delusions			
Typical ADNC	1.24	0.95 – 1.61	0.11
PART-low	1.03	0.75 – 1.40	0.86
PART-high	1.01	0.69 – 1.49	0.94
Hallucinations			
Typical ADNC	1.18	0.90 – 1.55	0.23
PART-low	0.91	0.66 – 1.26	0.59
PART-high	0.96	0.64 – 1.44	0.85
Agitation or aggression			
Typical ADNC	1.06	0.93 – 1.21	0.41
PART-low	0.9	0.77 – 1.06	0.21
PART-high	0.97	0.80 – 1.18	0.76
Depression or dysphoria			
Typical ADNC	0.98	0.75 – 1.28	0.89
PART-low	1.08	0.79 – 1.49	0.62
PART-high	0.93	0.63 – 1.38	0.71
Anxiety			
Typical ADNC	1.19	0.99 – 1.43	0.060
PART-low	0.96	0.77 – 1.19	0.69
PART-high	1.05	0.80 – 1.38	0.70
Elation or euphoria			
Typical ADNC	1.05	0.96 – 1.14	0.31
PART-low	0.98	0.88 – 1.08	0.64
PART-high	0.99	0.87 – 1.12	0.86
Apathy or indifference			
Typical ADNC	**1.25**	**1.01 – 1.54**	**0.037**
PART-low	0.97	0.76 – 1.25	0.83
PART-high	1.16	0.85 – 1.58	0.35
Disinhibition			
Typical ADNC	1.01	0.85 – 1.20	0.92
PART-low	0.82	0.66 – 1.01	0.061
PART-high	0.87	0.67 – 1.13	0.30

OR = odds ratio; CI = confidence interval
AP-ADNC = Thal phase 4-5 and Braak NFT stage 0-2, Typical ADNC = Thal phase 4-5 and Braak NFT stage 3-5, PART-low = Thal phase 0 and Braak NFT stage 0-2, PART-high = Thal phase 0 and Braak NFT stage 3-4
*AP-ADNC group was the reference group.
** Adjusted for the number of *APOE* ε4, age at death, and sex

Supplementary Table 6. (Continued)

Group*	Adjusted**		
	OR	95 % CI	P-value
Irritability or lability			
Typical ADNC	1.11	0.97 – 1.28	0.12
PART-low	0.99	0.85 – 1.17	0.95
PART-high	1	0.82 – 1.22	0.99
Motor disturbance			
Typical ADNC	1.07	0.87 – 1.32	0.51
PART-low	0.94	0.73 – 1.20	0.61
PART-high	1.04	0.77 – 1.42	0.78
Nighttime behaviors			
Typical ADNC	**0.67**	**0.46 – 0.97**	**0.036**
PART-low	0.80	0.51 – 1.25	0.33
PART-high	0.86	0.49 – 1.49	0.59
Appetite and eating problems			
Typical ADNC	0.85	0.71 – 1.00	0.055
PART-low	**0.79**	**0.64 – 0.96**	**0.021**
PART-high	0.82	0.64 – 1.05	0.12

OR = odds ratio; CI = confidence interval
AP-ADNC = Thal phase 4-5 and Braak NFT stage 0-2, Typical ADNC = Thal phase 4-5 and Braak NFT stage 3-5, PART-low = Thal phase 0 and Braak NFT stage 0-2, PART-high = Thal phase 0 and Braak NFT stage 3-4
*AP-ADNC group was the reference group.
** Adjusted for the number of *APOE* ε4, age at death, and sex

Supplementary Table 7. Genetic associations of 84 nucleotide polymorphisms in the multinomial regression analyses.

Variant	Chr	Gene	Position	Effect Allele	AF	AD OR	Typical-ADNC			PART-low			PART-high		
							OR	*SE*	*P*	OR	*SE*	*P*	OR	*SE*	*P*
rs141749679	1	*SORT1*	109345810	C	0.01	1.38	0.23	1.23	0.23	-	-	-	-	-	-
rs679515	1	*CR1*	207577223	T	0.34	1.13	0.77	0.22	0.24	0.58	0.28	**0.045**	0.61	0.33	0.14
rs72777026	2	*ADAM17*	9558882	G	0.27	1.06	1.65	0.32	0.12	1.72	0.36	0.13	1.55	0.42	0.29
rs17020490	2	*PRKD3*	37304796	C	0.28	1.06	1.24	0.28	0.45	1.32	0.32	0.39	1.13	0.39	0.76
rs143080277	2	*NCK2*	105749599	C	0.01	1.47	-	-	-	-	-	-	-	-	-
rs6733839	2	*BIN1*	127135234	T	0.66	1.17	1.40	0.19	0.077	1.14	0.22	0.55	1.70	0.26	**0.041**
rs139643391	2	*WDR12*	202878716	T	0.23	0.94	0.71	0.25	0.16	0.90	0.29	0.71	0.68	0.36	0.28
rs10933431	2	*INPPSD*	233117202	G	0.43	0.93	0.80	0.21	0.27	0.92	0.24	0.73	0.89	0.29	0.69
rs16824536	3	*MME*	155069722	A	0.11	0.92	0.56	0.34	0.086	0.60	0.42	0.22	0.54	0.51	0.22
rs61762319	3	*MME*	155084189	G	0.04	1.16	1.68	0.72	0.47	1.40	0.82	0.68	1.21	0.98	0.85
rs115435541[*]	4	*IDUA*	990916	A	0.09	0.95	1.23	0.46	0.65	0.95	0.56	0.92	1.64	0.58	0.40
rs6846529	4	*CLNK*	11023507	C	0.48	1.07	1.11	0.21	0.61	0.89	0.24	0.63	0.83	0.29	0.51
rs2245466	4	*RHOH*	40197226	G	0.54	1.05	0.87	0.19	0.45	0.82	0.23	0.39	0.60	0.28	0.069
rs112403360	5	ANKH	14724304	A	0.14	1.09	1.12	0.36	0.75	0.81	0.44	0.64	0.99	0.50	0.98
rs62374257	5	*COX7C*	86927378	C	0.40	1.07	1.39	0.24	0.16	1.35	0.27	0.27	1.37	0.32	0.32
rs871269	5	*TNIP1*	151052827	T	0.54	0.96	0.78	0.19	0.20	0.77	0.23	0.26	1.43	0.26	0.16
rs113706587	5	*RASGEF1C*	180201150	A	0.21	1.09	0.68	0.27	0.15	0.53	0.33	0.057	0.38	0.45	**0.032**
rs6605556	5	*HLA-DQA1*	32615322	G	0.32	0.91	0.94	0.23	0.80	0.94	0.27	0.81	0.78	0.34	0.47
rs10947943	6	*UNC5CL*	41036354	A	0.23	0.94	1.36	0.29	0.29	1.46	0.33	0.25	2.07	0.36	**0.044**
rs188904277[*]	6	*UNC5CL*	41053109	A	0.03	1.41	0.74	0.77	0.70	0.76	0.94	0.77	0.63	1.24	0.71
rs60755019	6	*TREM2*	41181270	G	0.03	1.55	0.37	0.71	0.16	0.43	0.85	0.32	0.39	1.18	0.43
rs7767350	6	*CD2AP*	47517390	T	0.49	1.08	0.80	0.19	0.22	0.60	0.23	**0.030**	0.83	0.27	0.48
rs785129	6	*HS3ST5*	114291731	T	0.60	1.04	0.87	0.20	0.47	0.83	0.23	0.41	0.83	0.27	0.51
rs6943429	7	*UMAD1*	7817263	T	0.67	1.05	1.08	0.19	0.69	1.10	0.22	0.67	0.91	0.26	0.72
rs10952097	7	*ICA1*	8204382	T	0.22	1.07	0.88	0.28	0.66	1.24	0.32	0.50	1.12	0.38	0.76
rs13237518	7	*TMEM106B*	12229967	A	0.69	0.96	1.11	0.19	0.59	0.90	0.22	0.61	1.63	0.26	0.057
rs2189966[*]	7	*JAZF1*	28132447	C	0.43	0.95	0.76	0.20	0.18	0.86	0.24	0.52	0.74	0.29	0.30
rs6966331	7	*EPDR1*	37844191	T	0.58	0.96	1.00	0.19	0.99	0.99	0.22	0.96	0.98	0.27	0.93

AD OR is the odds ratio of Alzheimer's disease reported by Bellenguez et al. AP-ADNC is the reference group.

Supplementary Table 7. (Continued)

Variant	Chr	Gene	Position	Effect Allele	AF	AD OR	Typical-ADNC			PART-low			PART-high		
							OR	*SE*	*P*	OR	*SE*	*P*	OR	*SE*	*P*
rs76928645	7	*SEC61G*	54873635	T	0.17	0.93	1.02	0.30	0.96	0.80	0.36	0.54	0.60	0.47	0.28
rs7384878	7	*SPDYE3*	100334426	C	0.48	0.92	0.98	0.20	0.91	0.96	0.24	0.85	0.72	0.29	0.26
rs11771145	7	*EPHA1*	143413669	A	0.57	0.95	1.12	0.20	0.57	1.07	0.23	0.76	1.26	0.27	0.39
rs1065712	8	*CTSB*	11844613	C	0.10	1.09	0.62	0.36	0.19	0.69	0.44	0.40	0.91	0.51	0.85
rs73223431	8	*PTK2B*	27362470	T	0.59	1.07	0.95	0.19	0.81	0.95	0.22	0.80	1.07	0.26	0.80
rs11787077	8	*CLU*	27607795	T	0.63	0.91	0.84	0.18	0.34	0.94	0.22	0.79	1.48	0.26	0.13
rs34173062	8	*SHARPIN*	144103704	A	0.15	1.13	1.44	0.38	0.34	1.33	0.43	0.51	1.81	0.49	0.22
rs1800978	9	*ABCA1*	104903697	G	0.25	1.06	1.07	0.27	0.79	1.18	0.31	0.60	0.47	0.45	0.093
rs7912495	10	*USP6NL*	11676714	G	0.72	1.06	1.15	0.19	0.46	0.95	0.22	0.83	1.01	0.26	0.97
rs7068231	10	*ANK3*	60025170	T	0.63	0.95	0.84	0.18	0.34	0.84	0.22	0.43	0.76	0.26	0.29
rs6586028	10	*TSPAN14*	80494228	C	0.33	0.93	0.97	0.24	0.89	0.98	0.28	0.95	1.05	0.33	0.89
rs6584063	10	*BLNK*	96266650	G	0.07	0.89	1.55	0.60	0.46	2.56	0.64	0.14	1.45	0.76	0.63
rs7908662	10	*PLEKHA1*	122413396	G	0.70	0.96	0.76	0.18	0.13	0.93	0.21	0.73	0.97	0.25	0.91
rs10437655	11	*SPI1*	47370397	A	0.63	1.06	0.97	0.18	0.89	0.95	0.21	0.83	0.84	0.26	0.49
rs1582763	11	*MS4A4A*	60254475	A	0.57	0.91	1.00	0.19	0.99	1.02	0.23	0.93	0.90	0.27	0.68
rs3851179	11	*EED*	86157598	T	0.56	0.9	1.18	0.20	0.41	1.21	0.23	0.40	1.40	0.27	0.21
rs74685827	11	*SORL1*	121482368	G	0.04	1.19	1.11	0.73	0.88	0.77	0.91	0.78	1.18	0.98	0.87
rs11218343	11	*SORL1*	121564878	C	0.09	0.84	0.58	0.40	0.16	0.91	0.45	0.83	0.81	0.56	0.71
rs6489896	12	*TPCN1*	113281983	C	0.13	1.08	1.78	0.44	0.19	0.98	0.52	0.96	0.70	0.67	0.60
rs17125924	14	*FERMT2*	52924962	G	0.16	1.1	0.75	0.31	0.36	0.62	0.39	0.22	0.52	0.50	0.19
rs7401792	14	*SLC24A4*	92464917	G	0.63	1.04	1.39	0.20	0.10	1.38	0.23	0.17	1.46	0.27	0.16
rs12590654	14	*SLC24A4*	92472511	A	0.56	0.93	0.77	0.19	0.17	0.92	0.22	0.72	0.70	0.27	0.19
rs7157106	14	*IGH gene cluster*	105761758	A	0.55	1.05	1.68	0.21	**0.015**	1.31	0.25	0.27	1.99	0.28	**0.013**
rs10131280	14	*IGH gene cluster*	106665591	A	0.25	0.94	1.75	0.33	0.089	2.76	0.36	**0.0048**	2.23	0.41	**0.048**
rs8025980	15	*SPPL2A*	50701814	G	0.57	0.96	1.27	0.20	0.22	1.55	0.23	0.054	1.35	0.27	0.26
rs602602	15	*MINDY2*	58764824	A	0.50	0.94	0.99	0.20	0.97	1.12	0.24	0.64	1.13	0.28	0.66
rs117618017	15	*APH1B*	63277703	T	0.25	1.11	1.09	0.29	0.76	1.41	0.33	0.29	1.48	0.38	0.29
rs3848143	15	***SNX1***	64131307	G	0.42	1.05	2.32	0.28	**0.0025**	2.28	0.31	**0.0076**	2.80	**0.34**	**0.0026**

AD OR is the odds ratio of Alzheimer's disease reported by Bellenguez et al. AP-ADNC is the reference group.

Supplementary Table 7. (Continued)

Variant	Chr	Gene	Position	Effect Allele	AF	AD OR	Typical-ADNC			PART-low			PART-high		
							OR	*SE*	*P*	OR	*SE*	*P*	OR	*SE*	*P*
rs12592898	15	*CTSH*	78936857	A	0.25	0.94	0.85	0.25	0.50	0.79	0.30	0.42	1.02	0.34	0.95
rs1140239	16	*DOC2A*	30010081	T	0.62	0.94	0.92	0.18	0.65	1.07	0.22	0.76	0.93	0.26	0.78
rs889555	16	*BCKDK*	31111250	T	0.48	0.95	1.07	0.20	0.75	1.17	0.24	0.51	1.14	0.28	0.64
rs4985556	16	*IL34*	70660097	A	0.20	1.07	1.23	0.32	0.52	1.23	0.37	0.58	1.33	0.43	0.51
rs450674	16	*MAF*	79574511	C	0.60	0.96	0.72	0.19	0.080	1.12	0.22	0.59	0.75	0.26	0.27
rs12446759	16	*PLCG2*	81739398	G	0.64	0.95	1.05	0.19	0.81	1.22	0.22	0.38	1.01	0.26	0.96
rs72824905	16	*PLCG2*	81908423	G	0.01	0.74	0.28	0.85	0.13	0.30	1.24	0.33	-	-	-
rs16941239	16	*FOXF1*	86420604	A	0.07	1.13	0.86	0.52	0.77	1.32	0.57	0.62	0.83	0.75	0.81
rs56407236	16	*PRDM7*	90103687	A	0.14	1.11	0.60	0.29	0.081	0.71	0.35	0.33	0.34	0.53	**0.041**
rs35048651	17	*WDR81*	1728046	T	0.38	1.06	1.18	0.24	0.48	1.23	0.28	0.45	0.98	0.33	0.96
rs7225151	17	*SCIMP*	5233752	A	0.24	1.08	0.90	0.27	0.69	0.83	0.32	0.57	0.43	0.45	0.056
rs2242595	17	<u>*MYO15A*</u>	18156140	A	0.22	0.94	0.73	0.25	0.21	0.85	0.30	0.57	0.33	0.46	**0.016**
rs5848	17	*GRN*	44352876	T	0.52	1.07	1.13	0.20	0.55	1.16	0.23	0.52	1.11	0.27	0.70
rs199515	17	***WNT3***	46779275	G	0.35	0.94	0.64	0.21	**0.031**	0.74	0.25	0.24	0.75	0.29	0.33
rs616338	17	*ABI3*	49219935	T	0.02	1.32	1.75	1.04	0.59	1.30	1.24	0.83	0.96	1.43	0.98
rs2526377	17	*TSPOAP1*	58332680	G	0.69	0.95	0.85	0.18	0.38	0.84	0.21	0.41	1.07	0.25	0.79
rs4277405	17	*ACE*	63471557	C	0.61	0.94	1.30	0.20	0.19	1.16	0.23	0.53	1.28	0.27	0.36
rs12151021	19	*ABCA7*	1050875	A	0.56	1.1	1.39	0.20	0.10	0.78	0.24	0.32	1.18	0.28	0.54
rs149080927	19	*KLF16*	1854254	G	0.73	1.05	0.93	0.18	0.68	0.87	0.21	0.53	1.16	0.25	0.56
rs429358	19	***APOE***	44908684	G	0.49	-	1.94	0.24	**0.0059**	0.21	0.37	**2.5×10^{-5}**	-	-	-
rs7412	19	***APOE***	44908822	C	0.28	-	0.33	0.32	**5.5×10^{-4}**	1.06	0.35	0.86	1.27	0.40	0.54
rs9304690	19	*SIGLEC11*	49950060	T	0.40	1.05	0.83	0.22	0.39	0.74	0.26	0.25	0.96	0.30	0.88
rs587709	19	*LILRB2*	54267597	C	0.54	1.05	1.18	0.19	0.39	0.97	0.23	0.91	1.04	0.27	0.88
rs1358782	20	*RBCK1*	413334	A	0.41	0.95	0.77	0.20	0.19	0.76	0.24	0.26	0.60	0.30	0.091
rs6014724	20	*CASS4*	56423488	G	0.17	0.89	0.64	0.28	0.11	0.91	0.33	0.77	0.65	0.42	0.30
rs6742	20	*SLC2A4RG*	63743088	T	0.38	0.95	1.20	0.23	0.43	1.36	0.27	0.25	0.79	0.34	0.48
rs2154481	21	*APP*	26101558	T	0.75	0.95	0.86	0.18	0.41	0.94	0.22	0.77	1.33	0.26	0.27
rs2830489	21	*ADAMTS1*	26775872	T	0.47	0.95	0.88	0.20	0.53	0.94	0.23	0.80	0.66	0.29	0.16

AD OR is the odds ratio of Alzheimer's disease reported by Bellenguez et al. AP-ADNC is the reference group.

Supplementary Table 8. Genetic associations with group in the multinomial regression analyses (AP-ADNC vs. others). Typical ADNC included Braak stage VI.

Variant	Gene	Typical-ADNC			PART-low			PART-high		
		OR*	*SE*	*P*	OR*	*SE*	*P*	OR*	*SE*	*P*
rs679515	*CR1*	0.77	0.22	0.22	0.57	0.28	**0.040**	0.61	0.33	0.14
rs6733839	*BIN1*	1.37	0.19	0.095	1.17	0.23	0.48	1.67	0.26	0.051
rs113706587	*RASGEF1C*	0.66	0.26	0.11	0.53	0.33	0.059	0.37	0.45	**0.027**
rs10947943	*UNC5CL*	1.24	0.28	0.45	1.46	0.33	0.25	2.02	0.36	0.050
rs7767350	*CD2AP*	0.80	0.19	0.23	0.60	0.23	**0.030**	0.82	0.27	0.45
rs7157106	*IGH gene cluster*	1.59	0.21	**0.025**	1.30	0.24	0.28	2.04	0.28	**0.010**
rs10131280	*IGH gene cluster*	1.79	0.32	0.070	2.63	0.36	**0.0066**	2.21	0.40	**0.049**
rs3848143	*SNX1*	2.26	0.27	**0.0029**	2.33	0.31	**0.0057**	2.77	0.34	**0.0028**
rs56407236	*PRDM7*	0.60	0.29	0.075	0.70	0.36	0.32	0.34	0.53	**0.042**
rs2242595	*MYO15A*	0.71	0.24	0.16	0.86	0.29	0.60	0.34	0.46	**0.017**
rs199515	*WNT3*	0.64	0.20	**0.026**	0.71	0.25	0.27	0.77	0.29	0.36
rs429358	*APOE*	2.31	0.37	**3.7×10^{-4}**	0.22	0.37	**4.3×10^{-5}**	-	-	-
rs7412	*APOE*	0.32	0.31	**2.1×10^{-4}**	1.11	0.35	0.76	1.19	0.40	0.66

AP-ADNC is the reference group. $P < 0.05$ is highlighted with bold letters.

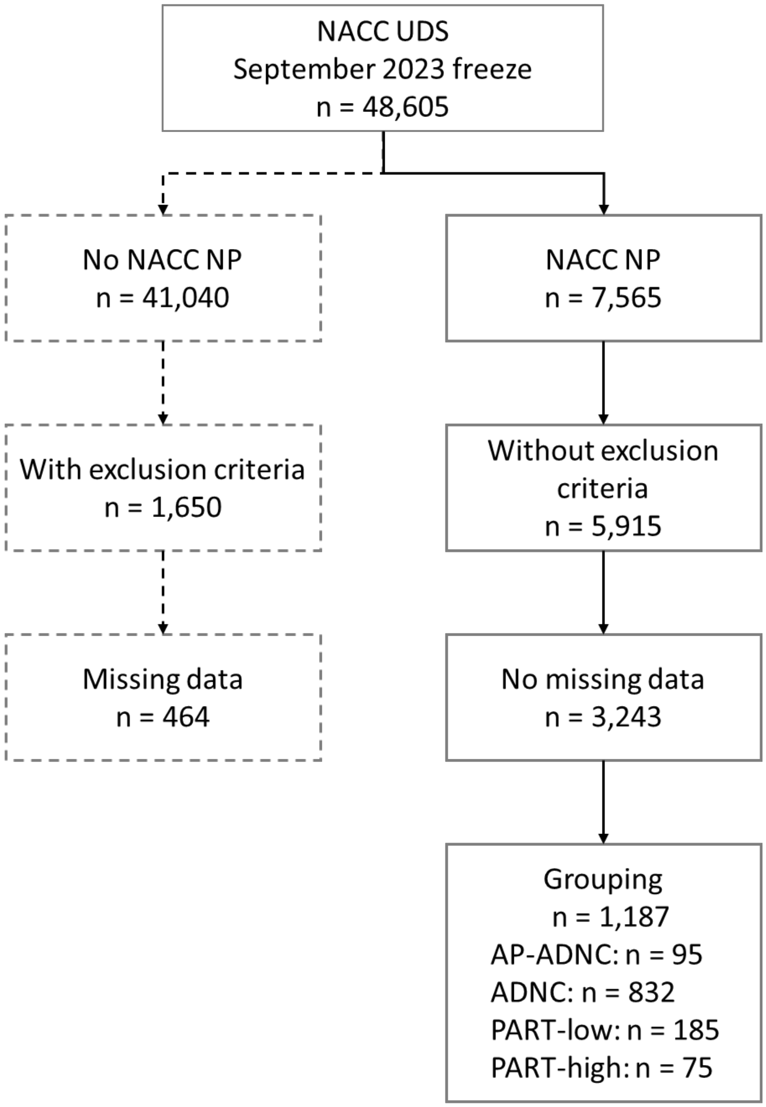

Supplementary Fig. 1. Flow diagram of the subjects included in the analyses.

NACC = National Alzheimer's Coordinating Center; NP = Neuropathology; UDS = Uniform Data Set

The exclusion criteria and grouping scheme are in Supplementary Table 1 and Supplementary Table 2, respectively.

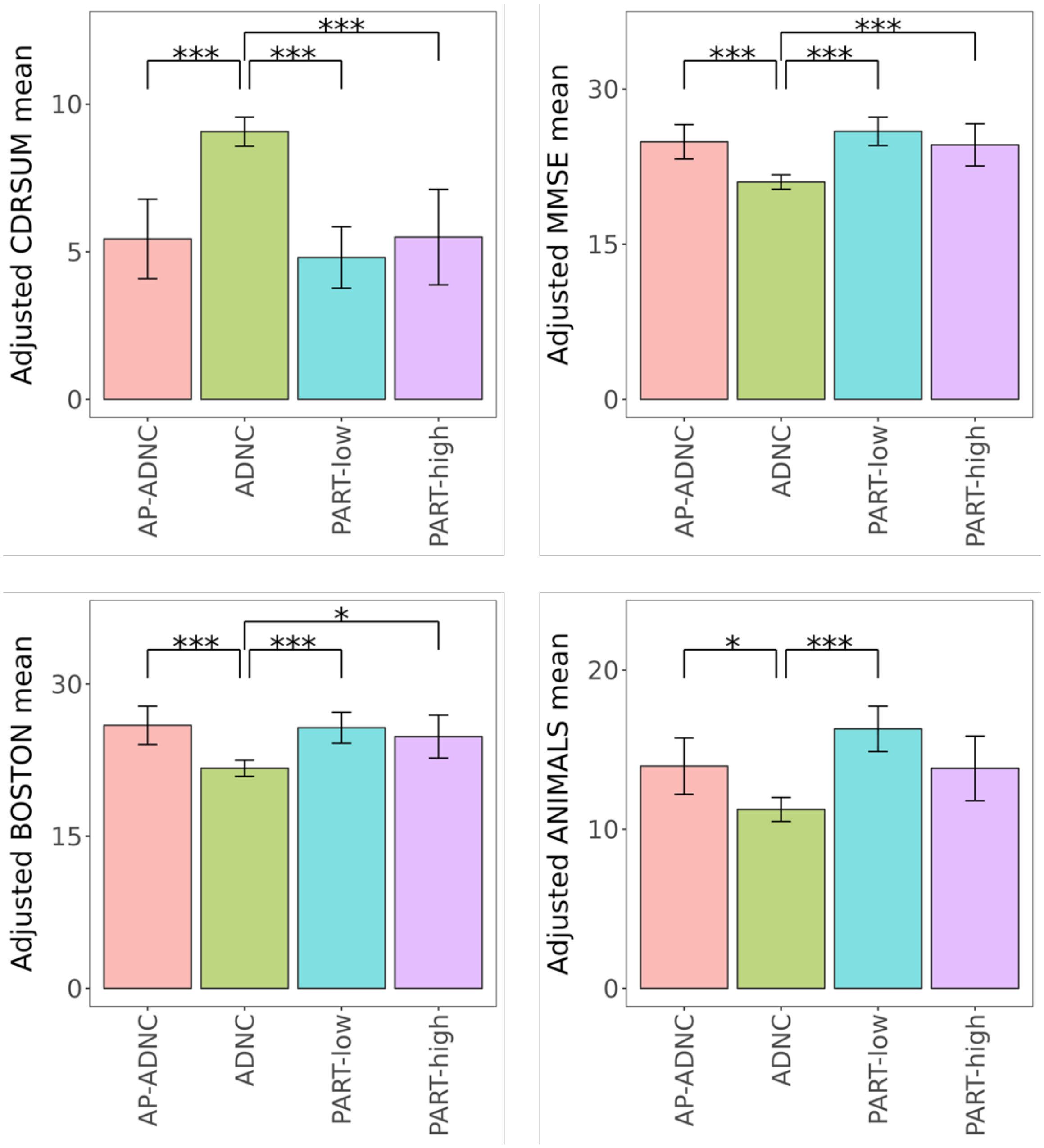

Supplementary Fig. 2. Adjusted means of cognitive test score measured at the last visit within three years before death by groups.

*P<0.05, **P<0.01, and ***P<0.001
CDRSUM = CDR® sum of boxes; MMSE = Mini Mental State Examination; BOSTON = Boston Naming Test; ANIMALS = Total number of animals naming
AP-ADNC = Thal phase 4-5 and Braak NFT stage 0-2, Typical ADNC = Thal phase 4-5 and Braak NFT stage 3-5, PART-low = Thal phase 0 and Braak NFT stage 0-2, PART-high = Thal phase 0 and Braak NFT stage 3-4

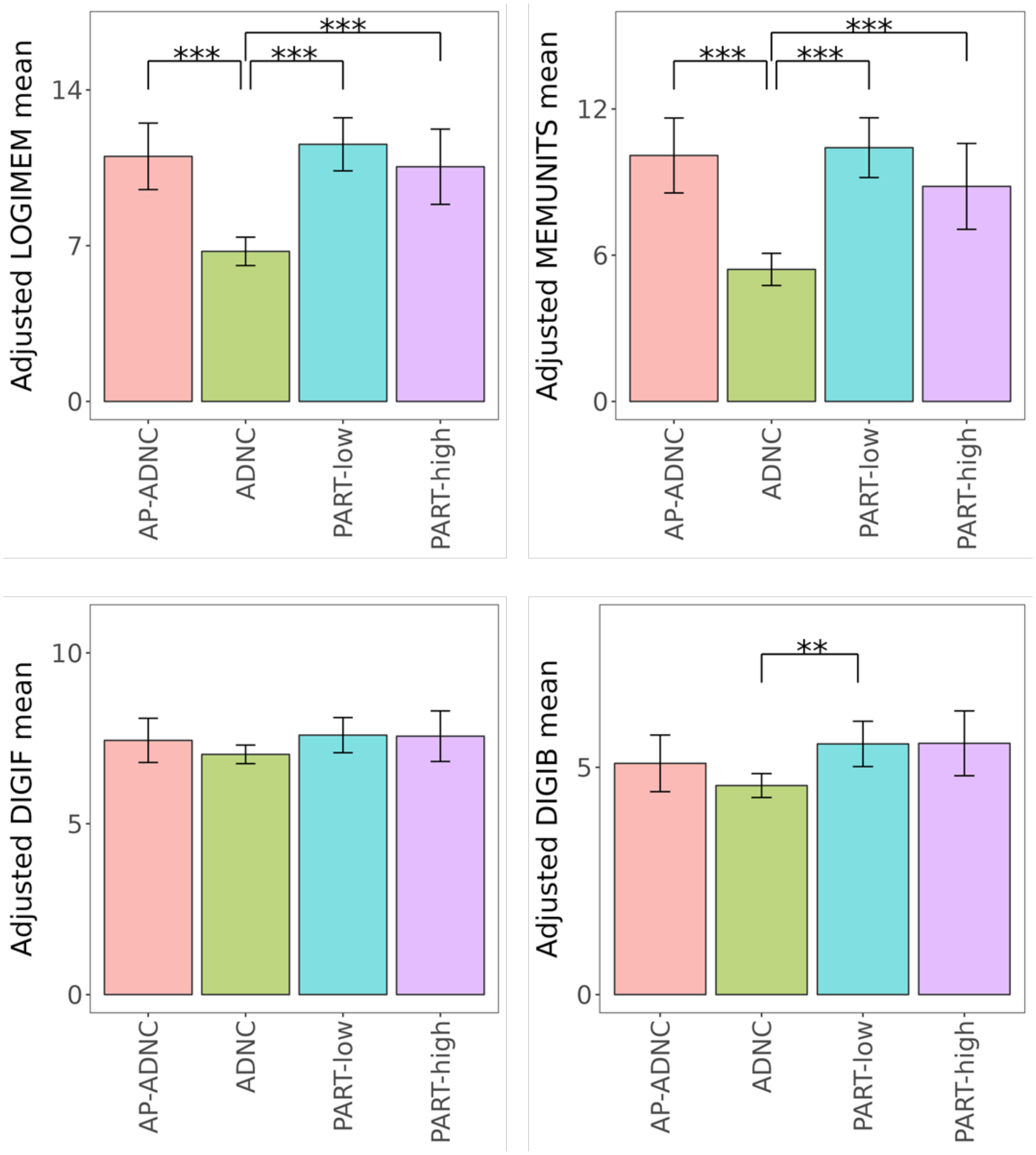

Supplementary Fig. 2. (Continued)

*P<0.05, **P<0.01, and ***P<0.001
LOGIMEM = Wechsler Memory Scale-Revised (WMS-R) Logical Memory – immediate; MEMUNITS = Wechsler Memory Scale-Revised (WMS-R) Logical Memory – delayed; DIGIF = Digit span forward trials correct; DIGIB = Digit span backward trials correct
AP-ADNC = Thal phase 4-5 and Braak NFT stage 0-2, Typical ADNC = Thal phase 4-5 and Braak NFT stage 3-5, PART-low = Thal phase 0 and Braak NFT stage 0-2, PART-high = Thal phase 0 and Braak NFT stage 3-4
